# Prenatal prognostic factors for isolated right congenital diaphragmatic hernia: a single center’s experience

**DOI:** 10.1186/s12887-021-02931-6

**Published:** 2021-10-20

**Authors:** Jiyoon Jeong, Byong Sop Lee, Teahyen Cha, Euiseok Jung, Ellen Ai-Rhan Kim, Ki-Soo Kim, Dae Yeon Kim, Jung-Man Namgoong, Seong Chul Kim, Mi-Young Lee, Hye-Sung Won

**Affiliations:** 1 Department of Pediatrics, University of Ulsan College of Medicine, Asan Medical Center Children’s Hospital, 88 Olympic-ro 43-gil, Songpa-gu, Seoul, 05505 Korea; 2 Department of Pediatric Surgery, University of Ulsan College of Medicine, Asan Medical Center Children’s Hospital, Seoul, Korea; 3 Department of Obstetrics and Gynecology, University of Ulsan College of Medicine, Asan Medical Center, 88 Olympic-ro 43-gil, Songpa-gu, Seoul, 05505 Korea

**Keywords:** Congenital diaphragmatic hernia, Right-sided congenital diaphragmatic hernia, Observed-to-expected lung area-to-head circumference ratio, Extracorporeal membrane oxygenation

## Abstract

**Background:**

Right-sided congenital diaphragmatic hernia (RCDH) is relatively rare compared with left-sided congenital diaphragmatic hernia (LCDH). Clinical data of RCDH, especially with respect to antenatal prediction of neonatal outcome, are lacking. The aim of this study was to report the treatment outcomes of patients with antenatally diagnosed RCDH and to evaluate the predictability of observed-to-expected lung area-to-head circumference ratio (O/E LHR) for perinatal outcomes, focused on mortality or extracorporeal membrane oxygenation (ECMO) requirement.

**Methods:**

We retrospectively reviewed the medical records of newborn infants with isolated RCDH. We analyzed and compared the clinical and prenatal characteristics including the fetal lung volume, which was measured as the O/E LHR, between the survivors and the non-survivors.

**Results:**

A total of 26 (66.7%) of 39 patients with isolated RCDH survived to discharge. The O/E LHR was significantly greater in survivors (64.7 ± 21.2) than in non-survivors (40.5 ± 23.4) (*P* =.027). It was greater in survivors without ECMO requirement (68.3 ± 15.1) than non-survivors or those with ECMO requirement (46.3 ± 19.4; *P* = .010). The best O/E LHR cut-off value for predicting mortality in isolated RCDH was 50.

**Conclusions:**

The findings in this study suggest that O/E LHR, a well-characterized prognostic indicator in LCDH, could be applied to a fetus with antenatally diagnosed RCDH. A large cohort study is required to verify the association between O/E LHR values and the graded severity of RCDH.

## Introduction

Congenital diaphragmatic hernia (CDH) is a serious congenital anomaly with a high mortality rate. The incidence of CDH is 1 in 2500 to 4000 live births, with most cases being left-sided CDH (LCDH) [1]. The key medical treatment strategy for CDH is the appropriate respiratory and hemodynamic support for pulmonary arterial hypertension during the early postnatal days. Unfortunately, some patients who are unresponsive to medical treatment eventually require extracorporeal membrane oxygenation (ECMO) support [2]. The survival rate of CDH has been reported to be higher in the ECMO centers than in non-ECMO centers [3]. Therefore, identifying prenatal risk factors to predict early postnatal outcome and determining the requirement for ECMO support in the fetus diagnosed with CDH are important.

Several antenatal parameters have been found to aid in predicting the outcome of CDH. Liver herniation into intrathoracic area and the degree of lung hypoplasia, as estimated by the observed-to-expected lung area-to-head circumference ratio (O/E LHR), have been suggested as prognostic indicators [4–8]. However, most of these parameters are detected in patients with LCDH. Whether these can be considered as risk factors for right-sided CDH (RCDH), which accounts for only 10–15% of all CDH cases, is unclear [9]. We hypothesized that O/E LHR, a proven prognostic indicator in patients with LCDH, would also be applicable in patients with RCDH. The aim of this study was to report the treatment outcomes of patients with antenatally diagnosed RCDH and to evaluate the predictability of O/E LHR for perinatal outcomes, focused on mortality or ECMO requirement.

## Method

### Study population and data selection

We reviewed the medical records of patients diagnosed with CDH who were born and hospitalized in the neonatal intensive care unit in Asan Medical Center, Seoul, Korea, between January 2006 and October 2020. The eligibility criterion for inclusion was prenatal diagnosis of RCDH. The exclusion criteria were newborns with additional congenital anomalies, such as major structural malformation, chromosomal, and/or single gene disorders (i.e., non-isolated CDH) [10]. We analyzed the following demographic and clinical characteristics of all patients: gestational age at birth; body measurements such as birth weight; sex; mode of delivery; 1- and 5-min APGAR scores; total hospital days; maternal age; use of high frequency oscillatory ventilator (HFOV) and inhaled nitric oxide (iNO); associated major structural or chromosomal anomalies; repair operation; grade of defects [11]; patch repair; requirement for ECMO support; and survival [10]. This study was approved by the Institutional Review Board (IRB) of Asan Medical Center, South Korea (IRB No. 2020-1916). No specific ethical consent was required for a retrospective analysis.

### Imaging evaluation

The standard ultrasound imaging of the fetus suspected of CDH was performed at our center for a detailed evaluation to confirm the diagnosis and to exclude the presence of additional structural anomalies. Ultrasound measurements were performed using A30, WS80A (Samsung Medison Co., Ltd, Seoul, Korea), Voluson E8, or E10 Expert (General Electric Healthcare Austria GmbH & Co. OG, Zipf, Austria) with a 2–6-MHz transabdominal probe. Data on O/E LHR was available since 2014 and all were obtained in the second and/or third trimesters of pregnancy by two experienced obstetricians (MY Lee and HS Won) working at the fetal treatment center in our hospital during the study period. The lung area contralateral (left) to RCDH was originally measured by two different methods: the product of the two longest perpendicular linear measurements of the lung (O/E LHR longest) and manual tracing of the limits of the lung (O/E LHR trace) measured at the level of the four-chamber view of the heart on a transverse scan of the fetal thorax. The head circumference (mm) was retrieved from medical records. The lung area-to-head circumference ratio (LHR) was calculated as the lung area divided by the head circumference [7, 12]. The O/E LHR was calculated as described by Jani et al*.* and expressed as the percentage of the expected mean for the gestational age at the time of evaluation as the O/E LHR [6]. Herniated organs were identified by visual assessment on ultrasound images.

### Management protocols for infants with RCDH

All infants who were prenatally diagnosed with CDH were intubated in the delivery room at birth and admitted to the neonatal intensive care unit. In all cases, immediate ventilator support was initiated and maintained according to the local protocol. HFOV was indicated if the target preductal saturation (85–95%) or partial pressure of arterial carbon dioxide (45–60 mmHg) was not achieved by a conventional ventilator with a high peak inspiratory pressure (up to 25 to 30 cmH_2_O) and respiratory rate (> 40–60/min).

The criteria for neonatal ECMO were as follows: 1) infants with a gestational age of ≥ 34+0 weeks or birth weight ≥ 2,000 g without lethal congenital malformations, severe intracranial hemorrhage or brain injury, 2) severe cardiorespiratory failure evidenced by (1) oxygenation index > 40, (2) failure to wean from 100% oxygen, (3) echocardiographic evidence of severe pulmonary hypertension and/or cardiac dysfunction, or (4) pressure resistant hypotension, and/or shock despite maximal cardiopulmonary support (i.e., iNO and inotropes). Echocardiography was performed in all cases of antenatally diagnosed CDH at least within 4 hours after birth by pediatric cardiologists. The echocardiographic diagnosis of pulmonary hypertension was made by means of the following criteria: 1) flattened or displaced ventricular septum, 2) right-to-left shunting through the patent ductus arteriosus and/or foramen ovale, and 3) increased right ventricular pressure using peak tricuspid regurgitant jet velocity estimated by the modified Bernoulli equation. The severity of pulmonary hypertension was classified as moderate (estimated right ventricle pressure > 2/3 systemic pressure) or severe (≥ systemic pressure). Pressor-resistant hypotension was defined by persistent low mean arterial pressure less than gestational age in weeks despite volume expansion, inotropics, and hydrocortisone.

Surgical repair of CDH was performed at least after 48 hours of life to allow for clinical stabilization and a fall in pulmonary vascular resistance. The surgical approach, by either open (transthoracic or transabdominal) or endoscopic surgery (thoracoscopic or laparoscopic), was at the discretion of the pediatric surgeon after evaluation of clinical characteristics, including patient size and echocardiographic evaluation.

In our center, treatment protocol of CDH, including high-frequency ventilator use, inotropic use, iNO, and “a delayed repair protocol” did not change during the study period. ECMO has been available since 2008. The policy of ECMO running and surgical repair, especially in patients requiring ECMO, evolved during the study period. A team approach aimed at improving the survival rate of CDH patients under ECMO was started in 2018. The main shift was the change in surgical approach from “on-ECMO repair” to “off-ECMO repair”. However, if the patient failed to wean from ECMO after 14 to 21 days of life, repair on ECMO was attempted.

### Data analysis and statistics

The primary outcome measure was mortality. To evaluate the predictability of O/E LHR for perinatal outcomes, mortality and ECMO requirement were analyzed as separate outcome measures. We analyzed and compared the demographic and prenatal ultrasound findings, such as the lung volume measured as the O/E LHR between the subgroups using one-way analysis of variance (see below). The effect of the O/E LHR on outcomes was assessed using the Fisher’s exact test and Mann–Whitney U test. Receiver operating characteristic (ROC) curves were used to determine the cut-off value of the O/E LHR for mortality and mortality or ECMO requirement. Data are presented as the area under the curve (AUC) and 95% confidence interval (CI). The cut-off value was calculated using the Youden index. Statistical analyses were performed using IBM SPSS statistics ver. 20.0 (IBM Co., Armonk, NY, USA). Categorical data are presented as numbers (percentages), and continuous data are presented as means ± standard deviations or medians. A *P* value of < .05 was considered statistically significant.

## Results

A total of 45 patients with RCDH were found to be eligible for the study. However, 6 of 45 patients were excluded because of non-isolated CDH (*n* = 6). Thus, 39 patients were included in the analysis. The median gestational age and birth weight were 38+0 weeks (range: 26+5 to 40+1 weeks) and 3040 g (range: 660–3587 g), respectively. Of the 39 patients, 26 (66.7%) patients survived to hospital discharge. Among the 13 patients (33.3%) who succumbed, 5 were on ECMO support and 8 did not receive ECMO. The indications for ECMO exclusion were extreme prematurity (gestational age < 30 weeks, *n* = 3), evidence of severe brain injury before ECMO (*n* = 3), and chronic lung disease after CDH repair (*n* = 2). In total, 31 (79.5%) patients underwent diaphragmatic repair. Almost all patients had liver herniation (*n* = 38, 97.4%) into the thorax. Other herniated organs were the bowel (*n* = 21, 53.8%), kidney (*n* = 3, 7.7%), stomach (*n* = 1, 2.5%), gall bladder (*n* = 1, 2.5%), and spleen (*n* = 1, 2.5%).

The 1-minute and 5-minute APGAR scores were higher and the incidence of mediastinal shifting and use of HFOV and iNO were lower in survivors than in non-survivors. Other intraoperative findings such as defect size and the rate of patch repair did not differ between the subgroups of mortality and in the other subgroups (Table 1).

**Table 1:** Perinatal characteristics and clinical outcomes in survivors and non-survivors

	Survivors (*n* = 26)	Non-survivors (*n* = 13)	*P* value
Male sex	19 (73.1)	6 (46.2)	.157
Cesarean section	18 (69.2)	10 (76.9)	.719
Gestational age, wk	37.6 ± 1.6	35.2 ± 4.6	.087
Birth weight, g	3025.3 ± 409.3	2795.2 ± 876.7	.735
1-min APGAR score	5 (3-8)	4 (0-7)	.034
5-min APGAR score	7 (5-9)	6 (3-8)	.016
Total hospital days	33.9 ± 14.0	37.7 ± 67.1	.058
Maternal age, year	31.5 ± 4.4	31.5 ± 3.0	.758
Head circumference, cm	33.7 ± 6.0	33.6 ± 4.7	.508
Chest circumference, cm	32.0 ± 2.2	30.3 ± 4.7	.691
Abdominal circumference, cm	28.4 ± 2.8	26.9 ± 4.6	.566
Polyhydramnios	13 (50.0)	7 (53.8)	.821
Pleural effusion	5 (19.2)	5 (38.5)	.235
Mediastinal shifting	16 (61.5)	13 (100)	.016
High frequency ventilator use	14 (53.8)	13 (100)	.003
Inhaled nitric oxide use	11 (42.3)	13 (100)	<.001
Patch repair^a^	13 (54.2)	4 (80)	.370
Defect size^a^			.194
A/B/C/D	2 / 5 / 14 / 2	0 / 1 / 2 / 2	

Values are presented as means ± standard deviations or numbers with percentages or ranges in parentheses.

*APGAR* Appearance, Pulse, Grimace, Activity, and Respiration

^a^ Data on the defect size were not available for some patients who did not undergo repair

The O/E LHRs data were available for 23 patients. Since there was a strong correlation between the O/E LHR longest and the O/E LHR trace (*r* = .917; *P* < .001), the O/E LHR trace was described as O/E LHR in this study. Patients were divided into the 4 subgroups according to mortality and ECMO requirement (Fig. . 1A). The O/E LHR was lower in non-survivors (40.5 ± 23.4) than in survivors (64.7 ± 21.2) (*P* = .027) (Fig. . 1B). In addition, the O/E LHR was significantly lower in the subgroup of mortality or ECMO requirement (46.3 ± 19.4) than in the other subgroups (68.3 ± 15.1) (*P* = .010) (Fig. . 1C).

**Fig. 1:**
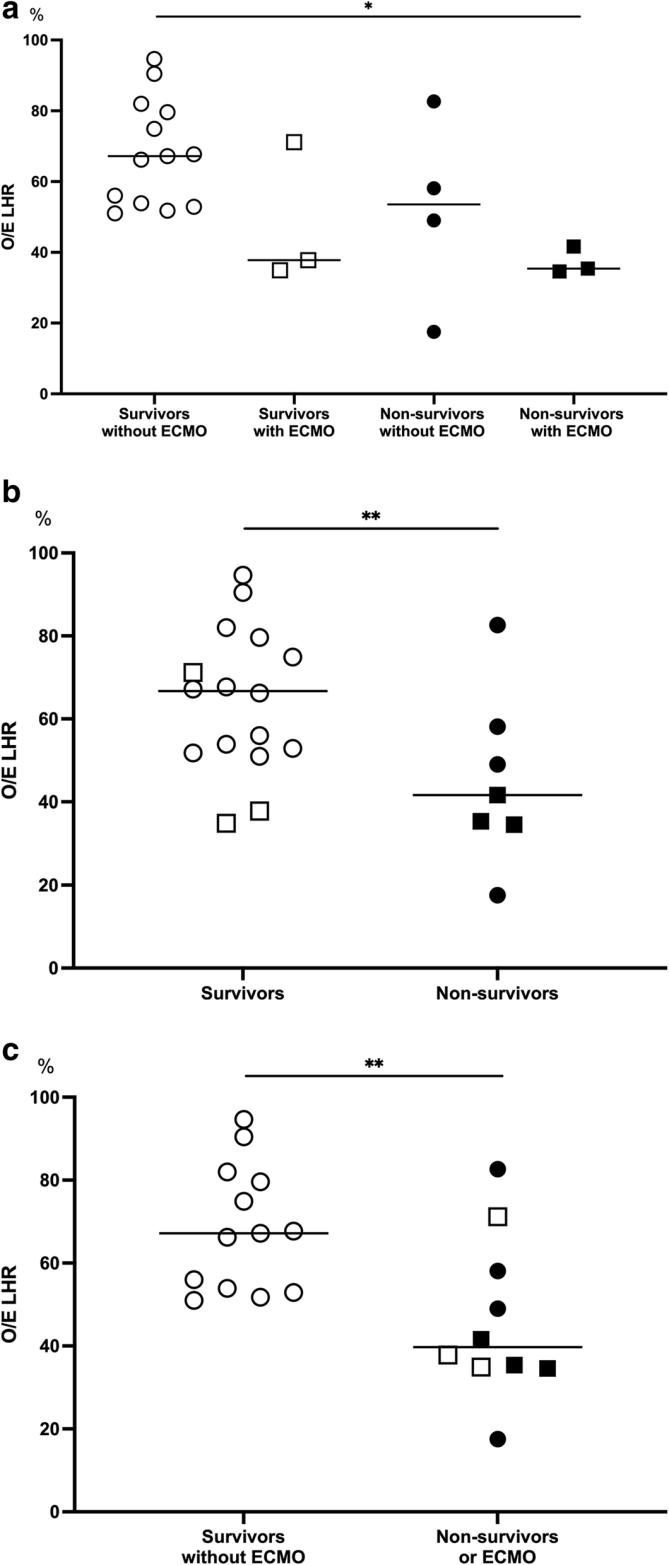
The O/E LHR and clinical outcomes depending on mortality and ECMO requirement: survivors without ECMO (Group A, open circles), survivors with ECMO (Group B, open rectangles), non-survivors without ECMO (Group C, closed circles), and non-survivors with ECMO (group D, closed rectangles). The O/E LHR was compared among the 4 subgroups (**A**), survivors (**A**, **B**) versus non-survivors (**C**, **D**) (**B**), and survivors without ECMO (**A**) versus non-survivors or ECMO (**B**, **C**, **D**) (**C**). Long midline bars indicate the median value in each group, respectively. * *P* < .005, ** *P* < .05. O/E LHR, observed-to-expected lung area-to-head circumference ratio; ECMO, extracorporeal membrane oxygenation

To determine whether the O/E LHR, a well described prenatal prognostic indicator of LCDH, can be considered a prognostic indicator of RCDH, patients were divided into two groups: patients with an O/E LHR of < 45 (*n* = 9) and those with an O/E LHR ≥45 (*n* = 14). The mortality was significantly higher in patients with an O/E LHR < 45 (6/9, 66.7%) than in those with an O/E LHR ≥ 45 (1/14, 7.1%; OR, 26.000; 95% CI, 2.219–304.702; *P* = .005). In addition, the mortality or ECMO requirement was significantly higher in patients with an O/E LHR < 45 (8/9, 88.9%) than in those with an O/E LHR ≥ 45 (2/14, 14.3%; OR, 48.000; 95% CI, 3.704–621.998; *P* = .001). The area under the ROC curve of the O/E LHR for predicting mortality in RCDH was 0.768 (95% CI, 0.527–1.000; *P* = .045) and mortality or ECMO requirement in RCDH was 0.815 (*P* = .011), which were statistically significant (Fig. . 2). The best O/E LHR cut-off value for predicting mortality (sensitivity: 87.5% and specificity: 71.4%) in isolated RCDH with a Youden index of 0.589 was 50%.

**Fig. 2:**
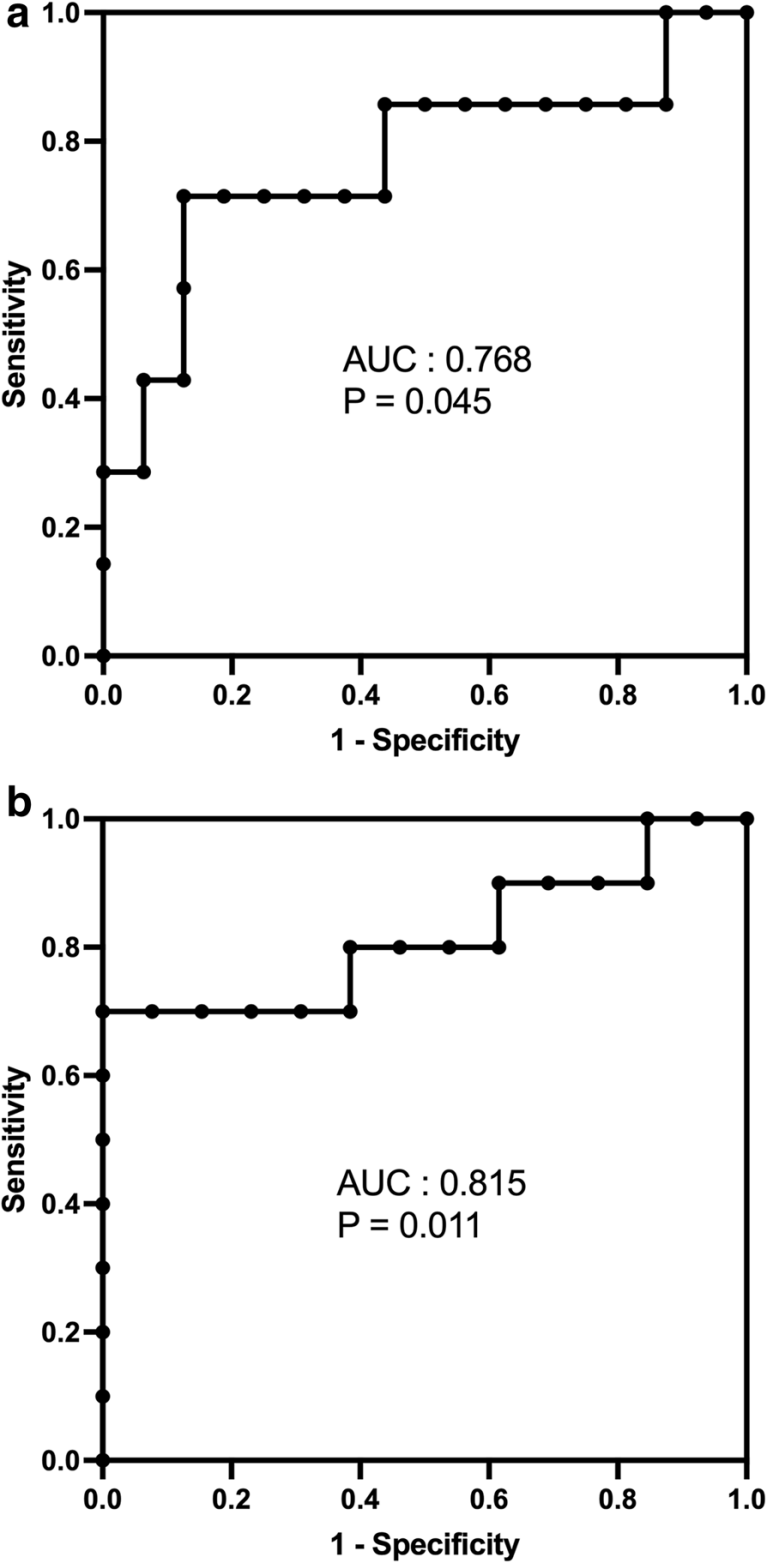
The ROC curve for predicting perinatal outcomes according to the O/E LHR. The ROC curve for predicting mortality (**A**) and mortality or ECMO requirement (**B**) in RCDH according to O/E LHR. AUC values were 0.768 (95% CI, 0.527–1.000; *P* = 0.045) and 0.815 (95% CI, 0.616–1.000; *P* = .011), respectively. ROC, receiver operating characteristic; O/E LHR, observed-to-expected lung area-to-head circumference ratio; AUC, area under the curve; ECMO, extracorporeal membrane oxygenation; CI, confidence interval

## Discussion

In the present study, the overall survival rate of isolated RCDH was 66.7%. The survival rate of RCDH in our study was similar to the range (29 - 78%) reported in previous large studies [12–17]. Controversy continues on whether the laterality of CDH affects mortality [15–21]. The uncertainty regarding the effect of laterality might be because of the small sample size, relative difficulty in prenatal diagnosis of RCDH, and postnatal management policies such as the timing of surgical repair. However, two recent large-scale studies in the United States found no difference in the overall mortality rates of RCDH and LCDH [18, 22]. In our previous report from a 27-year single-center cohort of CDH including non-isolated CDH, the survival rate of RCDH (60.5%) did not differ from that of LCDH (71.7%) (*P* = .182) [23]. Although the closure of the right pleuroperitoneal canal occurs before that of the left pleuroperitoneal canal, the mechanism of abdominal viscera herniation into the thoracic cavity resulting in the compression of the fetal lungs and leading to pulmonary hypoplasia is less likely to differ by the side [24]. Although a lower survival rate of RCDH (67%) versus LCDH (72%) was reported in a large international registry, the difference disappeared after controlling the diaphragmatic defect size [19]. The defect size, which could be observed only in patients who underwent surgical repair, did not differ between the survivors and non-survivors in our study. Overall, our results suggest that the defect laterality per se is not associated with mortality in isolated CDH.

In our study, the O/E LHR was a significant risk factor for isolated RCDH. The prognostic predictability of the O/E LHR was quite favorable, and the best O/E LHR cut-off value for mortality and mortality or requirement for ECMO support was 50%. This is consistent with the range of the predictive O/E LHR cut-off value (45–50%) in LCDH proposed by CDH EURO Consortium Consensus and antenatal CDH registry [25, 26]. The degree of pulmonary hypoplasia, as estimated by the LHR or O/E LHR, is known to be the most important determinant in predicting the outcome in infants with LCDH. A recent meta-analysis also demonstrated that the LHR and O/E LHR were significantly related to ECMO requirement in isolated LCDH [27]. However, whether the O/E LHR can predict survival in RCDH is debatable because the number of RCDH cases is relatively rare. Jani et al*.* demonstrated that the O/E LHR is useful for predicting subsequent survival in both LCDH and RCDH, in which no survival has been reported for an O/E LHR of < 25% [6]. DeKoninck et al*.* reported that after expectant in utero management, the survival rates in patients with RCDH and an O/E LHR of < 45 and < 30% were 17 and 0%, respectively [15]. In contrast, Victoria et al*.* raised questions regarding the reliability of the O/E LHR as a predictor of RCDH [14]. In this single-center study, the survival rate of RCDH was relatively high (up to 60%) even in patients with an O/E LHR of < 45%, probably because the results were derived from only five cases.

It remains unclear why the association between the measured O/E LHR and the prognosis of RCDH is inconsistent across the studies. It is possible that the laterality of the defect can influence the reliability of O/E LHR measure that is reflective of lung hypoplasia. Although a significant correlation between the 2D-measured O/E LHR and 3D-measured fetal lung volume was demonstrated in studies that used magnetic resonance (MR), this was not applicable to RCDH [6, 28]. The O/E LHR did not correlate with the actual fetal lung-to-body weight ratio in seven post-mortem cases of RCDH. It has been suggested that obtaining a reliable and reproducible four-chamber view to measure the contralateral lung area of RCDH could be more technically difficult relative to LCDH [6]. Research on the role of ipsilateral (right) lung volume in RCDH could be a topic of interest, although in LCDH, the visualization of ipsilateral lung measure could be infeasible in many cases, and the correlation with MR lung volume is relatively weaker than contralateral lung measure [29].

In the registry of isolated LCDH, the survival rate was reported based on the O/E LHR interval (15, 25, 35, and 45%) along with the presence of liver herniation [25]. A recent ECMO guideline for CDH proposed a prenatal risk stratification system, which includes an O/E LHR cut-off value of < 25%, liver herniation, and the O/E total lung volume [30]. However, it remains unclear whether such a graded classification can be used for RCDH. To the best of our knowledge, no study has investigated whether a dose–response relationship exists between the O/E LHR value and mortality or ECMO support requirement in RCDH with an O/E LHR of < 45%.

A well-known predictor of outcome in LCDH is the intrathoracic position of the liver [9, 26, 31]. However, the anatomical difference between left and right CDH makes the application of the liver status irrelevant because the liver is almost always up in every case of RCDH. Similar to previous reports, liver herniation was observed in almost all patients with RCDH [9, 14]. The degree of liver herniation, manifested by the percentage of the herniated liver and measured by fetal magnetic resonance imaging (MRI), was suggested as a prenatal indicator of LCDH [32]; however, the volume of the herniated liver was not found to be predictive of survival in a recent study on RCDH [14]. We had no data on the herniated liver volume because fetal MRI data were unavailable. Other fetal ultrasound findings, such as mediastinal shifting and polyhydramnios, were not associated with neonatal outcome in RCDH [33].

This study has some limitations. First, like other single center studies on LHR or O/E LHR in RCDH [12–14], the limitation of our study was its small sample size albeit it included patients examined during a 15-year time period. The prognostic role of the ultrasound measurement of contralateral lung in RCDH should be determined again in large studies by using multicenter or registry cohort. Interestingly, a multicenter study with a relatively large cohort of RCDH (*n* = 86) demonstrated that the O/E LHR >45% was associated with better survival in cases of expectant management [15].

Second, inherent to the retrospective design, inter-observer variation from the two obstetricians in the ultrasound examination could not be controlled. Despite this, the measurement was performed with the center-specific standard protocol and there was a strong correlation between the tracing method and the longest method. In this analysis, the O/E LHR trace was chosen for its better reproducibility [34]. Measurement of lung volumes by fetal MRI provide more objective data, albeit the controversy regarding predictability.

Third, during the study period, some of the management policy of CDH changed, including the timing of surgical repair in CDH with ECMO requirement, and this might have influenced the survival rate. However, the predictability of O/E LHR in RCDH did not differ in mortality and mortality or ECMO requirement. We expect to report more data in the near future and intend to provide a graded O/E LHR cut-off criterion.

## Conclusion

The findings in this study suggest that O/E LHR, a well-characterized prognostic indicator in LCDH, could be applied to a fetus with antenatally diagnosed RCDH. A large cohort study is required to verify the association between O/E LHR values and the graded severity of RCDH.

## Data Availability

The data that support the findings of this study are available from the corresponding author on reasonable request.

## Abbreviations

CDH: Congenital diaphragmatic hernia
ECMO: Extracorporeal membrane oxygenation
RCDH: Right-sided congenital diaphragmatic hernia
LCDH: Left-sided congenital diaphragmatic hernia
LHR: Lung area-to-head circumference ratio
O/E LHR: Observed-to-expected lung area-to-head circumference ratio
HFOV: High frequency oscillatory ventilator
iNO: Inhaled nitric oxide
ROC: Receiver operating characteristic
AUC: Area under the curve
CI: Confidence interval
MRI: Magnetic resonance imaging
